# Primary Biliary Cholangitis Pathogenesis: A Pathophysiology-Based Narrative Review

**DOI:** 10.3390/ijms27031388

**Published:** 2026-01-30

**Authors:** Klairi Papachristou, Maria Angelara, Konstantinos Manganas, Theodoros Androutsakos

**Affiliations:** 1 Department of Pathophysiology, Medical School, National and Kapodistrian University of Athens, 11527 Athens, Greece; klairi1papachristou@gmail.com (K.P.); mariaaggelara@gmail.com (M.A.); 2 First Department of Propaedeutic and Internal Medicine, “Laiko” Hospital, National and Kapodistrian University of Athens, 11527 Athens, Greece; kmagganas92@gmail.com

**Keywords:** autoimmune liver disease, primary biliary cholangitis, pathogenesis, liver, genetic factors, miRNAs, bicarbonate umbrella, immune dysregulation, bile acids

## Abstract

Primary biliary cholangitis (PBC) is a chronic, cholestatic disease, with a female predominance and a female-to-male ratio of approximately 10:1, that typically follows a slowly progressive, decades-long disease course. The disease is usually asymptomatic at the time of diagnosis and it is not uncommon for a patient to present with cirrhosis. Patients with PBC may also present with extrahepatic manifestations, including pruritus, chronic fatigue, and osteoporosis, while co-existence of other autoimmune diseases, such as autoimmune hepatitis, Hashimoto’s disease, Sjogren’s syndrome, or systemic sclerosis is not uncommon. The exact pathogenesis of PBC remains elusive with a variety of different factors, including genetic, epigenetic, and environmental ones, alongside immune dysregulation leading to a dysfunction of biliary “bicarbonate umbrella”, a protective mechanism by which cholangiocyte-secreted bicarbonate creates an alkaline microenvironment shielding the epithelium from bile acid-induced injury, and increased biliary epithelial cells apoptosis.

## 1. Introduction

**Figure 1:**
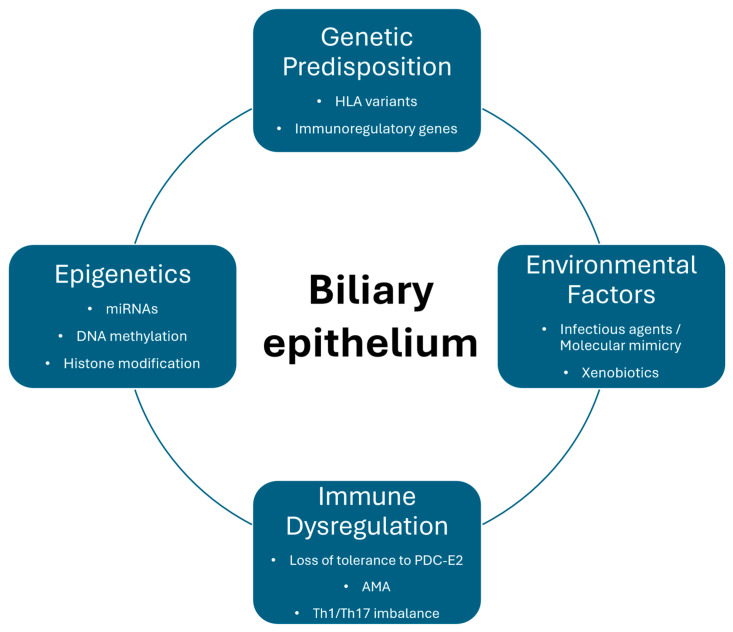
Key pathogenetic mechanisms contributing to biliary epithelial injury in primary biliary cholangitis. Environmental factors, epigenetic modifications, and immune dysregulation interact with genetic predisposition to disrupt bile acid homeostasis and compromise the biliary bicarbonate umbrella, culminating in the development of primary biliary cholangitis.

Primary biliary cholangitis (PBC) is a chronic autoimmune cholestatic liver disorder characterized by a Τ-cell lymphocyte-mediated destructive cholangitis that predominantly targets the biliary epithelial cells (BECs) of the small intrahepatic bile ducts [1,2]. The disease usually presents during the fifth decade of life and is strongly associated with female sex, with earlier reports showing a female to male ratio of almost 10:1, although, in recent literature, it has been demonstrated to be more prevalent in males than previously thought, with a female to male ratio closer to 4–6:1 [3,4]. The pathogenesis of the disease remains incompletely understood, even though environmental, genetic, and epigenetic factors are thought to contribute to disease development (Figure 1) [5,6,7,8]. A persistent elevation of cholestatic markers such as serum alkaline phosphatase (ALP), gamma glutamyl transferase (gGT), and bilirubin are the most common biochemical laboratory findings observed in PBC patients, often accompanied by an elevation in immunoglobulin-M concentration. Extrahepatic manifestations of PBC may include constitutional symptoms (such as fatigue and pruritus), autoimmune features (including sicca symptoms), metabolic complications (notably hyperlipidemia), and skeletal involvement such as osteoporosis [9,10,11]. Extrahepatic manifestations are observed in up to 73% of patients with PBC, with the most common being Sjögren’s syndrome, thyroid dysfunction, and systemic sclerosis. Patients with PBC are thought to be at increased risk of developing these extrahepatic manifestations, the majority of which are autoimmune in nature, reflecting a broader predisposition to autoimmunity [11]. The disease is often asymptomatic at the time of diagnosis, and it is not uncommon for patients to present with established cirrhosis, with clinical studies reporting that up to approximately half of patients had cirrhosis at study entry [12].

Autoantibodies play a key role in both the recognition and understanding of PBC. The vast majority of patients are positive for antimitochondrial antibodies (AMA), which target the E2 subunit of the pyruvate dehydrogenase complex (PDC-E2) and represent one of the most characteristic features of the disease. Nevertheless, a proportion of patients do not exhibit AMA positivity. In these cases, PBC-specific antinuclear antibodies, most commonly anti-glycoprotein 210 (anti-gp210) and anti-sp100, can be detected and are present in up to half of AMA-negative patients. Importantly, distinct autoantibody profiles in PBC appear to reflect underlying biological differences, with certain specificities—particularly anti-gp210—being associated with more aggressive disease phenotypes and poorer clinical outcomes [13,14,15]. Given the high specificity of these serological markers, current American and European guidelines support a diagnosis of PBC based on autoantibody positivity alone, without the need for routine liver biopsy [12,16].

Ursodeoxycholic acid (UDCA) has been the mainstay of treatment, even though almost 40% of patients fail to achieve biochemical response. In recent years, however, new treatment options based on disease pathogenesis, namely οbeticholic acid, seladelpar, and elafibranor, have emerged as adequate treatment alternatives [17].

## 2. Pathogenesis

The pathogenesis of PBC is largely unclear; however, evidence suggests that susceptibility to the disease results from the interplay of environmental triggers in patients with genetic predisposition [18]. The primary pathophysiological defect appears to involve disruption of the biliary bicarbonate umbrella, which is critical for protecting the bile ducts from harmful bile acids through the secretion of HCO_3_^−^ into the ductular lumen, a defect that seems to be common in a variety of different cholestatic diseases, including genetic cholestatic ones [19].

### 2.1. Genetic Factors

The importance of genetic risk in the development of the disease is highlighted by several complementary observations, including the notably high concordance rates seen in monozygotic twins as well as the progressively increased prevalence of the disease among first- through fifth-degree relatives of affected individuals [20,21]. However, while genetics are crucial, allelic variants most likely do not directly determine the disease but rather influence key biological processes that contribute to its development. Even though major advances have been made in defining the genetic architecture of PBC, particularly through large-scale genomic studies, many questions remain unanswered. In particular, the precise functional consequences of identified risk variants, as well as their mechanistic relevance to the disease process, remain insufficiently characterized and represent important areas for future research [22].

Genetic studies, particularly genome-wide association studies (GWAS), have identified several genetic loci regulating innate and adaptive immunity, many of which are associated with an increased risk of developing PBC. Among these loci, the most commonly found belong to the major histocompatibility complex (MHC) region [6]. More specifically, the presence of HLA-DR*08 alleles has been repeatedly associated with a heightened risk of PBC, whereas alleles such as HLA-DRB1*11 and HLA-DRB1*13 appear to confer a protective effect, reducing the likelihood of disease development [23]. However, considerable variation is observed among different ethnic groups. Studies involving European cohorts have identified HLA-DQA1*04:01, HLA-DQB1*04:02, and HLA-DRB1*08:01 as alleles predisposing to disease, whereas HLA-DQB1*03:01 was found to be protective. On the other hand, research conducted in Asian populations has highlighted different patterns of genetic susceptibility. In these groups, HLA-DRB1*08:03 has emerged as a notable risk allele, whereas HLA-DQB1*03:01, HLA-DQB1*06:04, and HLA-DRB1*13:02 have been associated with a decreased risk and are therefore considered protective [24].

Beyond the well-established contributions of human leukocyte antigen (*HLA*) genes, a growing body of evidence indicates that several non-HLA genes involved in the regulation of immune responses also play a meaningful role in the pathogenesis of PBC. Among these genes, the interleukin-12 (IL-12) pathway has attracted considerable attention. Multiple studies have demonstrated that variations in *IL12A*, *IL12RB2*, and *STAT4*—key genes responsible for mediating IL-12–dependent immune activation—are associated with altered susceptibility to the disease [20]. The IL-12 cytokine family itself, comprising IL-12, IL-23, IL-27, and IL-35, is central to the orchestration of immune responses. These cytokines contribute to immune regulation primarily by promoting the differentiation and activation of T helper 1 (Th1) cells, while concurrently inhibiting T helper 17 (Th17) cell activity through the induction of interferon-γ (IFN-γ) production. This balance between Th1 and Th17 pathways is critical for maintaining immune homeostasis, and disruptions in this system are believed to contribute to the development of autoimmune conditions such as PBC [25].

In addition to IL-12-related genes, several other genes implicated in immune regulation have been associated with PBC pathogenesis, including *IL7R*, which is involved in T cell survival and homeostasis; *IKZF3*, a transcription factor regulating lymphocyte differentiation; *CD80* and *CTLA4*, key modulators of T cell costimulatory signaling; and *NFKB1*, a central regulator of inflammatory and immune responses [6].

### 2.2. Epigenetic Regulation and microRNAs

Epigenetic mechanisms such as DNA methylation, histone modifications and non-coding RNA interference, have been proposed as a possible bridge between genetics and environmental factors influencing the onset and development of PBC [26].

MicroRNAs (miRNAs) are now widely recognized as key regulators within a broad spectrum of biological processes that influence both innate and adaptive immune responses. These small, non-coding RNA molecules play essential roles in controlling gene expression at the post-transcriptional level, thereby shaping immune cell development, differentiation, activation, and functional behavior. Through their ability to fine-tune signaling pathways, cytokine production, and inflammatory responses, miRNAs contribute to the maintenance of immune homeostasis and are considered to be important factors in the pathogenesis of various autoimmune and inflammatory diseases [27]. Padgett et al. [28], using miRNA array analysis, identified a total of 35 miRNAs that are differentially expressed in patients with end-stage PBC compared with healthy controls. Subsequent validation studies confirmed notable expression changes in a subset of these molecules, specifically demonstrating significant downregulation of miR-122a and miR-26a, along with upregulation of miR-328 and miR-299-5p in liver tissues from individuals with PBC (Table 1). The predicted and experimentally supported target genes of these dysregulated miRNAs are closely linked to key pathogenic processes implicated in PBC, including biliary inflammation, hepatocellular apoptosis, and reactive oxidative stress [28]. Another study conducted a bioinformatics analysis to investigate the potential functions of dysregulated miRNAs in peripheral B cells across different stages of PBC. The researchers reported that the expression levels of miR-223-3p and miR-21-5p in circulating B cells were significantly lower in advanced PBC than in early-stage disease [29].

Nevertheless, among the numerous miRNAs that have been investigated in the context of PBC, miR-506 currently stands out as the only one for which a clear and direct functional role in the immunopathogenesis of the disease has been demonstrated [30]. MiR-506 is upregulated in patients with PBC resulting in decreased anion exchanger 2 (AE2) and inositol-1,4,5-trisphosphate receptor type 3 (InsP3R3) expression [31,32]. AE2 is a Cl^−^/HCO_3_^−^ exchanger at the apical membrane of BECs that plays a key role in maintaining the so-called “biliary bicarbonate umbrella”. In addition, InsP3R3 is also essential for the preservation of the biliary bicarbonate umbrella through regulation of calcium release in cholangiocytes. Increased intracellular Ca^2+^ leads to Cl^−^ secretion via transmembrane 16A Cl^−^ channels (TMEM16A) making a Cl^−^ transmembrane concentration gradient which activates the Cl^−^/HCO_3_^−^ AE2 [33]. When miR-506 is overexpressed, its inhibitory effects on both AE2 and InsP3R3 disrupt this finely balanced system. Impaired bicarbonate secretion weakens the protective alkaline barrier, allowing bile acids to accumulate within cholangiocytes. Over time, this toxic buildup triggers cellular stress, promotes inflammatory signaling, and ultimately results in the apoptosis of biliary epithelial cells—one of the hallmark pathogenic events contributing to PBC progression [34].

**Table 1:** MiRNAs involved in PBC pathogenesis. This table summarizes microRNAs reported to be differentially expressed in patients with primary biliary cholangitis compared with controls. For each microRNA, the biological source analyzed (liver tissue, cholangiocytes, immune cell subsets, or peripheral blood) and the direction of expression change are indicated. Altered microRNA expression has been implicated in immune regulation, cholangiocyte injury, and fibrogenic pathways relevant to PBC pathogenesis.

MiRNA	Source	Expression	Reference
miR-506	Liver, cholangiocytes	Elevated	[31]
miR-328	Liver	Elevated	[28]
miR-299-5p	Liver	Elevated	[28]
miR-21	Liver, splenic CD4^+^, CD8^+^ T cells	Elevated	[35]
miR-210	Liver	Elevated	[36]
miR-132	Liver	Elevated	[37]
miR-34a	Liver	Elevated	[37]
miR-122a	Liver	Decreased	[28]
miR-26a	Liver	Decreased	[28]
miR-125b	Cholangiocytes	Decreased	[38]
miR-92a	Cholangiocytes	Decreased	[39]
miR-425	Peripheral CD4^+^ T cells	Decreased	[40]

### 2.3. Environmental Factors

Environmental factors are believed to play a pivotal role in triggering the autoimmune response in individuals with a genetic predisposition on PBC. A variety of environmental exposures, most notably infectious agents, xenobiotics, and other chemical compounds have been proposed to play a role in PBC pathogenesis though molecular mimicry. In particular, halogenated organic compounds and other xenobiotics, including metabolites of pharmaceuticals, anesthetic agents, and household or cosmetic chemicals, may chemically modify the lipoyl domain of the pyruvate dehydrogenase complex E2 subunit, generating neo-epitopes that mimic microbial antigens. These antigenic mimics may provoke immune responses that cross-react with mitochondrial autoantigens, thereby initiating the autoimmune cascade characteristic of PBC [41]. This mitochondria-targeting immune response leads to the exposure of mitochondrial antigens and subsequently the formation of AMA, which are highly specific disease biomarkers [22].

Various infections have been proposed as possible triggers to PBC. Among these, urinary tract infections—particularly those caused by *Escherichia coli*—have been repeatedly observed at higher rates in patients with PBC compared with the general population [42]. A potential explanation lies in the significant molecular similarity between PDC-E2, the primary epitope targeted by antimitochondrial antibodies, in humans and *E. coli*, which may result in cross-reactivity and a subsequent breakdown of immunological tolerance [43]. Another Gram-negative bacterium, *Novosphingobium aromaticivorans*, implicated in PBC pathogenesis, has two proteins that display a high level of homology with PDC-E2 and can induce a 1000-fold stronger reaction in PBC patients compared to *E. coli* [44]. In addition to these organisms, several other pathogens have been investigated as possible contributors to the disease process, including retroviruses, *Propionibacterium acnes*, *Chlamydia pneumoniae*, and *Lactobacillus delbrueckii* [22,42].

Xenobiotics—including compounds such as 2-octynoic acid and 2-nonynoic acid, which are commonly present in cosmetics, nail polishes, and various other personal care products—have also been implicated as potential environmental contributors to the development of PBC [45,46]. Although the precise mechanisms through which these substances might promote autoimmunity remain incompletely understood, several hypotheses have been proposed. These include the possibility that xenobiotics may chemically modify self-antigens, enhancing their immunogenicity, or that they may directly interfere with immune regulatory pathways, thereby facilitating a loss of tolerance. Despite these plausible mechanisms, current evidence remains limited and not yet definitive [47].

### 2.4. Immune Dysregulation

**Figure 2:**
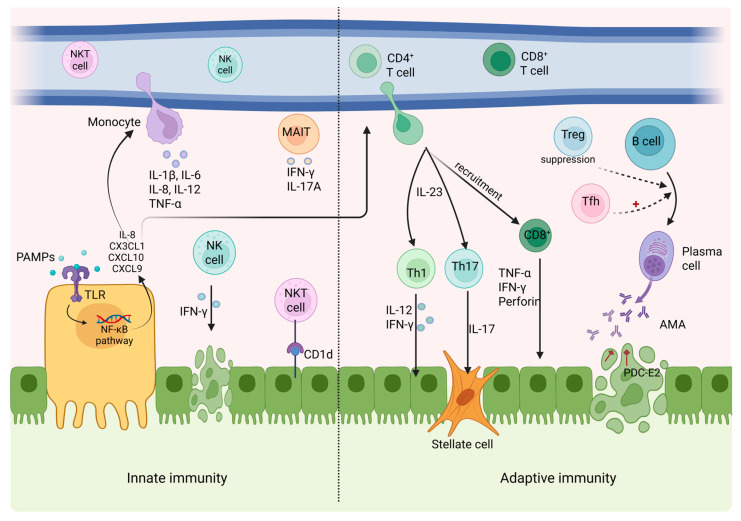
Chemokines targeting biliary epithelial cells. TLR-driven innate activation by PAMPs induces pro-inflammatory cytokines and chemokines that recruit monocytes and activate NK, NKT, and MAIT cells. On the adaptive side, responses involve IL-23-dependent Th1/Th17 differentiation and cytotoxic CD8^+^ T cell activation, leading to cholangiocyte injury. Tfh-mediated B cell activation and reduced Treg control promote AMA production against PDC-E2, collectively driving autoimmune bile duct damage. Solid arrows indicate direct interactions; dashed arrows indicate indirect, regulatory interactions and red plus signs denote activation. Created in https://BioRender.com.

The hallmark of PBC pathogenesis is the immune-mediated injury of BECs (Figure 2). This process is evident histologically by the dense infiltration of cytotoxic and inflammatory immune cells—such as CD4^+^ and CD8^+^ T lymphocytes, macrophages, B cells, and plasma cells, as well as natural killer (NK) and natural killer T (NKT) cells—surrounding and attacking the small and medium-sized intrahepatic bile ducts [48]. The cornerstone of this targeted immune response is the loss of tolerance to the mitochondrial autoantigen PDC-E2, the major target of antimitochondrial antibodies and autoreactive T cells in PBC [49].

#### 2.4.1. Autoantibody-Mediated Adaptive Immune Response

A hallmark immunological feature of PBC is the presence of antimitochondrial antibodies (AMA) directed against the E2 subunit of the pyruvate dehydrogenase complex. These autoantibodies reflect B cell activation and are closely linked to autoreactive T cell responses, which together contribute to cholangiocyte injury and disease perpetuation. As already mentioned, AMA, targeting PDC-E2, are found in the serum of >90% of patients with PBC. Although this mitochondrial autoantigen is present in all nucleated cells, the targeted biliary injury in PBC is thought to result from abnormal modification of mitochondrial PDC-E2 within apoptotic BECs. In contrast to normal apoptotic degradation, PDC-E2 in apoptotic BECs remains structurally preserved within distinct apoptotic blebs, retaining its immunogenic epitope in a form that is not fully denatured. These apoptotic bodies are subsequently taken up by antigen-presenting cells, including dendritic cells and macrophages, which process and present PDC-E2–derived peptides via MHC class II molecules. This process leads to activation of autoreactive CD4^+^ T cells and promotes B cell stimulation and production of antimitochondrial antibodies. The preservation of the PDC-E2 epitope allows circulating AMAs to readily recognize and bind to the exposed mitochondrial antigen. The resulting antigen–antibody complexes are thought to contribute directly to the localized autoimmune attack on the biliary epithelium, thereby promoting chronic inflammation and progressive ductal destruction [23,50,51]. Despite these insights, the initial triggers that lead to the loss of tolerance and the development of AMAs remain incompletely understood. Current theories suggest that molecular mimicry, as described above, plays a significant role. Additionally, chemical or structural modifications of PDC-E2, like oxidative modifications, altered acetylation, οr lipoylation, during cellular stress, infection, or xenobiotic exposure may create neo-antigens that further enhance immune recognition and drive the pathogenic immune response characteristic of PBC [51,52,53].

Nevertheless, 5–10% of PBC patients are AMA-negative, underlining the disease’s complexity. In recent years, a variety of antinuclear antibodies (ANA) have been implicated in PBC pathogenesis. PBC-specific ANAs are known to exhibit two characteristic immunofluorescence patterns that help distinguish them from ANA patterns seen in other autoimmune conditions: a perinuclear/rim-like membrane (RLM) pattern and a multiple nuclear dots (MND) pattern [54].

The anti–rim-like/membranous antibodies detected in PBC are predominantly directed against the nucleopore-associated protein gp210. In AMA-negative PBC patients, the reported anti-gp210 positivity rates range from 15.4% to 46.7%. Although specificity exceeds 99%, making anti-gp210 an excellent rule-in marker, sensitivity remains relatively low at around 25%, limiting its ability to detect all affected individuals [55]. Various mechanisms by which anti-gp210 antibodies contribute to the development and progression of PBC have been proposed; however, none of these provides a clear explanation [56]. Anti-gp210-positive patients with PBC tend to exhibit a more aggressive disease phenotype, with poorer response rates to UDCA, higher levels of biochemical markers of liver injury, and more advanced histopathological changes. Collectively, these features are associated with a less favorable overall prognosis, underscoring the significance of anti-gp210 as both a diagnostic and prognostic indicator in PBC [55].

The MND pattern is characterized by 3–20 discrete fluorescent dots distributed throughout the nucleus while sparing the nucleoli. This pattern arises from antibody reactivity against the nuclear body protein sp100, a 100-kDa antigen whose targeting is highly specific for PBC. They are detected in 8–44% of patients with PBC [54].

Meta-analytic data estimate a pooled sensitivity of 23.1% and a pooled specificity of 97.7%, highlighting anti-sp100 as a highly specific, though relatively insensitive, serological marker for the disease [57].

#### 2.4.2. Cellular Adaptive Immunity

In addition to the presence of highly specific AMAs in the serum of PBC patients, the central involvement of the adaptive immune system is further underscored by the prominent accumulation of autoreactive lymphocytes within the liver. Histological examinations consistently reveal dense infiltrates of PDC-E2-specific CD4^+^ and CD8^+^ T cells concentrated in the portal tracts, directly adjacent to the affected bile ducts. These T cells recognize and respond to the same mitochondrial autoantigen targeted by AMAs, providing strong evidence that both arms of the adaptive immune response—humoral and cellular—are actively engaged in driving biliary injury. Several chemokines are present in high concentrations within the portal tracts of patients with PBC, playing key roles in orchestrating the recruitment of autoreactive lymphocytes to the liver. In particular, CXCL10, CXCL9, and CX3CL1 are found abundantly in the portal area and thought to be the mediators of CD4^+^ and CD8^+^ T cell recruitment [51]. Moreover, BECs, apart from being targets, may function as APCs, by expressing MHC class I and II molecules, thereby amplifying local immune activation and promoting further T cell engagement [58]. Among the CD4^+^ T cell subtypes, T-helper 1 (Th1) cells seem to play a particularly prominent role in early disease [59]. Cytokines produced from Th1 such as IL-12 and IFN-γ, are considered of utmost importance in the disease’s onset, acting as drivers of the initial autoimmune response. However, as the disease progresses, a notable immunologic shift from a Th1-dominant environment to one in which the Th17 pathway becomes increasingly prominent is observed. This transition is facilitated by IL-23, a key cytokine that promotes the differentiation and expansion of Th17 cells [60]. IL-17, which is produced by Th17 cells, has been suggested to have pro-inflammatory and pro-fibrotic effects on PBC patients, including the stimulation of hepatic stellate cell proliferation, thus contributing to progressive fibrosis and liver damage [59,60,61].

T regulatory cells (Tregs) represent another T cell subpopulation that is responsible for preserving immunological self-tolerance and preventing the onset of autoimmune pathology. A relative decrease in the number of Tregs in peripheral blood has been noted in PBC patients, suggesting an impaired ability to restrain autoreactive immune responses [62]. Furthermore, studies have demonstrated a pronounced imbalance among the T follicular helper (Tfh) cells that regulate the differentiation of B cells, and T follicular regulatory (Tfr) cells that suppress the excessive or inappropriate immune response provoked by Tfh in patients with PBC. This dysregulation likely contributes to the break in self-tolerance, enhanced autoreactive B cell activation, and the production of disease-specific autoantibodies [63,64].

Finally, an increased CD4^+^ T cell-related, recruitment of CD8^+^ T cells to the liver has been found in patients with PBC. Once these CD8^+^ T cells are activated, they release a range of cytotoxic mediators that directly induce apoptosis of BECs, leading over time to the progressive destruction of small intrahepatic bile ducts, contributing to the chronic cholestasis and fibrotic remodeling characteristic of PBC [61].

#### 2.4.3. Innate Immunity

Apart from the well-established contribution of the adaptive immune system, innate immunity also seems to have a significant role in PBC pathogenesis. This is supported by several immunological features frequently observed in affected individuals, including the presence of granulomatous inflammation, elevated circulating levels of pro-inflammatory cytokines, increased polyclonal IgM, and an expansion of natural killer (NK) cells in peripheral blood [65]. As already mentioned, BECs can act as APCs through the expression of MHC-I and II. In addition, toll-like receptors (TLRs) expressed on the surface of biliary epithelial cells bind to various ligands, such as pathogen-associated molecular patterns (PAMPs); the expression of TLR2–5 and the TLR4 coreceptor MD-2, as well as the downstream signaling molecules MyD88 and IRAK-1, has been detected at the mRNA level in liver tissue, micro-dissected bile ducts, and cultured BECs. Activation of TLRs triggers downstream signaling via the nuclear factor κB (NF-κB) pathway, leading to the production and secretion of chemokines such as IL-8 and CX3CL1. These chemokines function as potent chemoattractants, promoting the recruitment of monocytes, CD4^+^ T cells, and CD8^+^ T cells to the portal tracts, thereby amplifying local inflammation [20,66,67,68]. Once activated, monocytes release pro-inflammatory cytokines (i.e., IL-1β, IL-6, IL-8, IL-12, and tumor necrosis factor alpha (TNF-α)), that perpetuate the adaptive immune response [69]. In parallel, macrophages respond to PDC-E2 contained within apoptotic blebs, as well as circulating AMAs, by secreting additional IL-12, thereby reinforcing Th1-mediated inflammation and contributing to the chronic immunopathology characteristic of PBC [70].

Natural killer cells (NK) are increased in the peripheral blood and the liver of PBC patients and demonstrate higher cytotoxic activity [61]. Furthermore, it has been reported that when NK cells interact with BECs, the IFN-γ produced by NK cells upregulates MHC-II expression on BECs. This aberrant expression promotes biliary damage by facilitating recognition and attack from autoreactive CD4^+^ T cells [71].

Natural killer T (NKT) cells are a subpopulation of T lymphocytes that share common properties with both T cells and NK cells and react with glycolipid antigens presented by CD1d [72]. Higher frequencies of CD1d-restricted NKT cells in the liver of PBC patients compared to peripheral blood have been documented, suggesting a potential contribution to local immune dysregulation [49].

Another important innate-like T cell subset relevant to PBC pathogenesis is the mucosal-associated invariant T (MAIT) cell population. MAIT cells reside abundantly around the bile ducts and secrete proinflammatory cytokines, including TNF-α, IFN-γ, and IL-17 [61]. The role of MAIT in PBC pathogenesis is highlighted by observations that indicate a reduced number of MAIT cells in the liver and blood of patients with PBC, exhibiting phenotypic features consistent with functional exhaustion. Moreover, stimulated by IL-12, MAIT cells produce IL-17A causing the expression of pro-fibrogenic genes in HSCs, thereby contributing to PBC-related fibrosis [73].

### 2.5. Biliary Epithelial Cells

#### 2.5.1. Bile Acids

PBC is marked by a profound disturbance in the synthesis, transport, and overall metabolism of bile acids (BAs). This dysregulation results in the accumulation of toxic bile acids within the liver parenchyma, ultimately leading to hepatocellular injury and cholangiocyte damage [20]. BAs are synthesized from cholesterol through two pathways. The classic (or neutral) pathway represents the primary route of BA synthesis in the liver and is initiated by the rate-limiting enzyme cholesterol-7α-hydroxylase (CYP7A1). In contrast, the alternative (or acidic) pathway begins with CYP27A1, which catalyzes the first step in a series of reactions that also lead to the formation of the two major primary bile acids: cholic acid and chenodeoxycholic acid. Once formed, these primary bile acids undergo conjugation with glycine or taurine, a modification that increases their water solubility and reduces their cytotoxicity. Conjugated bile acids are actively transported from hepatocytes into the bile canaliculi through the bile salt export pump (BSEP) and the multidrug resistance proteins, including ABCB1 and ABCC2 [74]. Consecutively, cholic and chenodeoxycholic acids are released in the small intestine in response to food, where they facilitate lipid digestion and are subsequently reabsorbed in the ileum by the enterocyte apical sodium-dependent bile acid transporter (ASBT). After uptake, these acids exit enterocytes through basolateral transporters such as the organic solute transporter αβ (OSTαβ) and return to the liver through the portal circulation, completing the enterohepatic cycle. A small fraction of BAs, approximately 5%, spill into colon, where they are metabolized by the gut microbiota and secondary bile acids deoxycholic acid and lithocholic acid (LCA) are generated [75].

Bile acids are not only detergents involved in lipid digestion but also mediators of signaling pathways that are involved in bile acid homeostasis through activation of the nuclear receptors farnesoid-X (FXR), pregnane-X (PXR) and vitamin D (VDR), and the membrane receptor TGR5 [76]. Through these receptors, bile acids finely modulate processes such as bile acid synthesis, transport, detoxification, inflammatory responses, and energy metabolism. In PBC, the pathological accumulation of BAs stimulates these receptors in order to enhance bile acid metabolism, reduce de novo synthesis, promote detoxification, and mitigate the cytotoxic effects of retained bile acids on hepatocytes and cholangiocytes [77].

The role of BAs in PBC pathogenesis is highlighted in numerous studies. In one of them, Trottier et al. showed that the primary BAs in the serum of PBC patients were 13.5-fold higher in comparison to non-cholestatic patients, with taurocholic acid (TCA) being the most abundant [78]. In addition, Chen et al. [79] reported an elevated serum BA concentration with a predominant accumulation of primary BAs in patients with newly diagnosed PBC. Importantly, the ratio of conjugated to unconjugated bile acids was substantially increased in both serum and fecal samples of PBC patients [79].

This primary and secondary BA imbalance seems to play a crucial role in PBC pathogenesis, since reduced serum levels of secondary BAs have been described in patients with PBC, indicating impaired intestinal biotransformation [79,80]. This observation is consistent with documented changes in the gut microbiota associated with PBC [81]. This altered equilibrium favors a more hydrophilic and less cytotoxic circulating bile acid pool which reflects a maladaptive response rather than restoration of physiological homeostasis [82]. Collectively, these findings suggest that microbiota-driven depletion of secondary bile acids in PBC contributes to disease progression by exacerbating immune dysregulation and compromising intestinal barrier integrity [80].

Another mechanism that facilitates BA’s secretion into urine is the sulfation. In cholestatic diseases, because of the distrurbed excretion of bile acids into the bile, the concentration of sulfated BAs in urine has been found to be higher [83]. Miura et al. reported that in PBC patients, the elevation of urinary sulfated bile acids correlates with progression to liver fibrosis [84].

Finally, an additional group of nuclear receptors involved in bile acid regulation are peroxisome proliferator-activated receptors (PPARs), which are activated by fatty acids. Upon activation, PPARs downregulate bile acid synthesis primarily through the suppression of CYP7A1 and CYP27A1, the key enzymes of the classic and alternative pathways of bile acid production, respectively. Given these mechanisms, pharmacologic PPAR agonists have emerged as promising therapeutic agents for PBC. Traditional fibrates, which activate PPAR-α, have demonstrated efficacy in improving cholestatic markers when used in combination with UDCA [85]. More recently, newer and more selective PPAR agonists—such as elafibranor (a dual PPAR-α/δ agonist) and seladelpar (a selective PPAR-δ agonist)—have shown significant clinical benefits in patients with PBC who have an inadequate response to UDCA and have recently received approval as second-line therapeutic options for the treatment of PBC [86,87].

#### 2.5.2. Biliary Bicarbonate Umbrella

**Figure 3:**
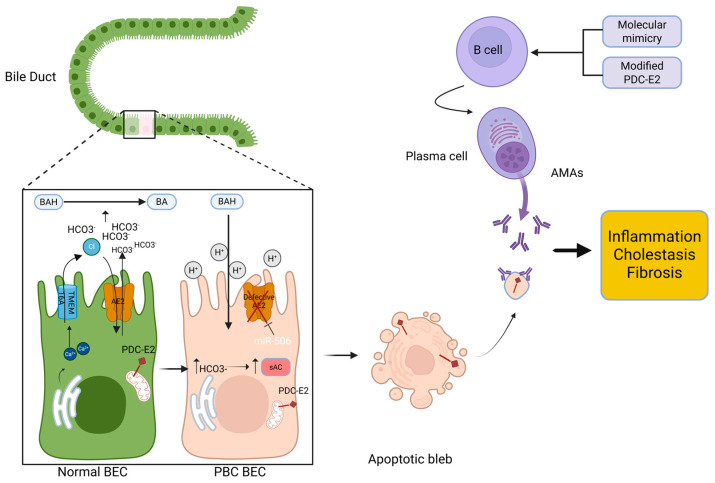
Biliary bicarbonate umbrella. In contrast to a healthy BEC, which is protected by the bicarbonate “umbrella” maintained by AE2 and TGR5, PBC BECs have impaired AE2 function, increased miR-506, and elevated sAC, reducing bicarbonate secretion and increasing susceptibility to bile acid injury. Damaged BECs release apoptotic blebs containing PDC-E2, triggering B cell activation and AMA production, which drives inflammation, cholestasis, and fibrosis. Created in https://BioRender.com.

BECs are safeguarded from bile acids by a protective mechanism known as the bicarbonate umbrella (Figure 3), which relies on the continuous secretion of HCO_3_^−^ into the lumen of the bile duct, creating an alkaline microenvironment that prevents protonation of BAs. The major contributor of this defense system is the AE2, a Cl^−^/HCO_3_^−^ exchanger, located on the apical surface of BECs. The hypothesis of a defective AE2 has long been implicated in PBC pathogenesis. Downregulation of AE2 results in a reduction of bicarbonate secretion leading to insufficient buffering capacity and the development of a more acidic luminal pH. Under these conditions, bile acids become protonated, which enhances their ability to enter cholangiocytes through passive diffusion [34,88,89]. Consequently, the intracellular pH becomes alkaline due to the accumulation of HCO_3_^−^, which is detected by soluble adenylyl cyclase (sAC), ultimately triggering apoptosis [90,91]. The cumulative effect of increased intracellular bile acid exposure, luminal acidification, and intracellular alkalinization creates a microenvironment conducive to biliary epithelial cell injury and death, thereby contributing to chronic cholestasis and progressive bile duct destruction. Experimental data for this model come from studies using Ae2(a,b)(-/-) mice which exhibit several hallmark features of human PBC, including AMA seropositivity and inflammatory portal infiltration [92].

#### 2.5.3. BEC Apoptosis and Senescence

As already mentioned, BECs play a critical role in PBC pathogenesis. Apart from their above-mentioned part in immune dysregulation, accumulating evidence indicates that BECs in PBC patients show increased susceptibility to apoptosis compared with those in other cholestatic liver diseases as well as higher levels of Fas, FasL, perforin, granzyme B, and TNF-related apoptosis-inducing ligands [30,93,94]. Under normal circumstances, within apoptotic cells, PDC-E2 binds to glutathione, rendering it immunologically inert. However, in PBC, this protective mechanism fails: PDC-E2 remains immunologically intact within the apoptotic blebs of BECs. This aberrant preservation allows AMAs to recognize and bind the exposed antigen, leading to the formation of immune complexes and subsequent activation of both innate and adaptive immune pathways [23,50,51].

Finally, senescence has emerged as another physiological process implicated in PBC. Senescent BECs—cells that have permanently exited the cell cycle in response to stress—produce a variety of pro-inflammatory cytokines and chemokines such as IL-6, IL-1, CX3CL1, CXCL8, and CCL2. These mediators promote the recruitment and retention of immune cells within the portal tracts, thereby amplifying local inflammation and accelerating the progression of biliary injury [95].

## 3. Conclusions

Advancements in understanding the complex pathogenesis of PBC have been instrumental in shifting treatment paradigms from symptomatic management toward targeted therapies aimed at slowing disease progression. Improved insights into cholestatic injury and bile acid homeostasis have facilitated the development of therapeutic agents targeting specific metabolic and nuclear receptor pathways, particularly the farnesoid X receptor and peroxisome proliferator-activated receptor signaling.

Based on these pathophysiological insights, several pharmacological approaches have been developed, with obeticholic acid, an FXR agonist, and the PPAR agonists elafibranor and seladelpar gaining regulatory approval in recent years. Ongoing research continues to explore combination strategies and novel bile acid-related targets, with the aim of improving biochemical response and long-term outcomes. Continued investigation of PBC pathogenesis remains essential for refining therapeutic strategies and enhancing patient care.

## Data Availability

No new data were created or analyzed in this study. Data sharing is not applicable to this article.

## Abbreviations

The following abbreviations are used in this manuscript:

PBC	Primary biliary cholangitis
BEC	Biliary epithelial cells
ALP	Alkaline phosphatase
gGT	Gamma glutamyl transferase
AMA	Antimitochondrial antibodies
PDC-E2	E2 subunit of the pyruvate dehydrogenase complex
Anti-gp210	Anti-glycoprotein 210
HCO_3_^−^	Bicarbonate
UDCA	Ursodeoxycholic acid
GWAS	Genome-wide association studies
MHC	Major histocompatibility complex
HLA	Human leukocyte antigen
IL-12	Interleukin-12
Th1	T helper 1
Th17	T helper 17
IFN-γ	Interferon-γ
MiRNAs	MicroRNAs
AE2	Anion exchanger 2
InsP3R3	1,4,5-trisphosphate receptor type 3
NK	Natural killer
NKT	Natural killer T
ANA	Antinuclear antibodies
RLM	Rim-like membrane
MND	Multiple nuclear dots
Tregs	T regulatory cells
Tfh	T follicular helper
Tfr	T follicular regulatory
TLRs	Toll-like receptors
PAMPs	Pathogen associated molecular patterns
NF-κΒ	Nuclear factor κΒ
TNF-α	Tumor necrosis factor alpha
MAIT	Mucosal-associated invariant T
BAs	Bile acids
CYP7A1	Cholesterol-7α-hydroxylase
BSEP	Bile salt export pump
ASBT	Apical sodium-dependent bile acid transporter
OSTαβ	Organic solute transporter αβ
LCA	Lithocholic acid
TCA	Taurocholic acid
FXR	Farnesoid-X receptors
PXR	Pregnane-X receptors
VDR	Vitamin D receptors
PPARs	Peroxisome proliferator-activated receptors
sAC	Soluble adenylyl cyclase
